# Supplementary material for: Pyruvate Dehydrogenase Kinase 1 inhibition mediated oxidative phosphorylation enhancement in cartilage promotes osteoarthritis progression
Source: BMC Musculoskelet Disord. 2023 Jul 20;24:597. doi: 10.1186/s12891-023-06585-6 (PMC10357736; doi:10.1186/s12891-023-06585-6)
Supplement: Supplementary file 1 — Additional file 1. [file 12891_2023_6585_MOESM1_ESM.zip › MF.Gsea.1653627316242/GOMF_ACTIVIN_BINDING.html]

Details for gene set GOMF\_ACTIVIN\_BINDING[GSEA]

|  || Dataset | 55457\_collapsed\_to\_symbols.cls.cls #OA\_versus\_normal.cls.cls #OA\_versus\_normal\_repos |
| Phenotype | cls.cls#OA\_versus\_normal\_repos |
| Upregulated in class | normal |
| GeneSet | GOMF\_ACTIVIN\_BINDING |
| Enrichment Score (ES) | -0.5117068 |
| Normalized Enrichment Score (NES) | -1.462488 |
| Nominal p-value | 0.08687943 |
| FDR q-value | 0.21575859 |
| FWER p-Value | 1.0 |
Table: GSEA Results Summary

  

Fig 1: Enrichment plot: GOMF\_ACTIVIN\_BINDING      
 Profile of the Running ES Score & Positions of GeneSet Members on the Rank Ordered List

  

| SYMBOL | TITLE | RANK IN GENE LIST | RANK METRIC SCORE | RUNNING ES | CORE ENRICHMENT || 1 | FKBP1A | FKBP prolyl isomerase 1A [Source:HGNC Symbol;Acc:HGNC:3711] | 2293 | 0.328 | -0.0944 | No |
| 2 | ACVR1 | activin A receptor type 1 [Source:HGNC Symbol;Acc:HGNC:171] | 3898 | 0.165 | -0.1769 | No |
| 3 | TGFBR1 | transforming growth factor beta receptor 1 [Source:HGNC Symbol;Acc:HGNC:11772] | 5280 | 0.050 | -0.2715 | No |
| 4 | ACVR2A | activin A receptor type 2A [Source:HGNC Symbol;Acc:HGNC:173] | 5960 | -0.002 | -0.3237 | No |
| 5 | ACVR1B | activin A receptor type 1B [Source:HGNC Symbol;Acc:HGNC:172] | 6368 | -0.033 | -0.3469 | No |
| 6 | TGFBR3 | transforming growth factor beta receptor 3 [Source:HGNC Symbol;Acc:HGNC:11774] | 6868 | -0.069 | -0.3682 | No |
| 7 | ENG | endoglin [Source:HGNC Symbol;Acc:HGNC:3349] | 7213 | -0.097 | -0.3702 | No |
| 8 | SMAD7 | SMAD family member 7 [Source:HGNC Symbol;Acc:HGNC:6773] | 7231 | -0.098 | -0.3466 | No |
| 9 | TGFBR2 | transforming growth factor beta receptor 2 [Source:HGNC Symbol;Acc:HGNC:11773] | 8241 | -0.174 | -0.3806 | No |
| 10 | FST | follistatin [Source:HGNC Symbol;Acc:HGNC:3971] | 8511 | -0.194 | -0.3519 | No |
| 11 | AMHR2 | anti-Mullerian hormone receptor type 2 [Source:HGNC Symbol;Acc:HGNC:465] | 10571 | -0.381 | -0.4147 | Yes |
| 12 | ACVR2B | activin A receptor type 2B [Source:HGNC Symbol;Acc:HGNC:174] | 11011 | -0.431 | -0.3390 | Yes |
| 13 | FSTL3 | follistatin like 3 [Source:HGNC Symbol;Acc:HGNC:3973] | 11853 | -0.560 | -0.2615 | Yes |
| 14 | ACVRL1 | activin A receptor like type 1 [Source:HGNC Symbol;Acc:HGNC:175] | 12242 | -0.656 | -0.1244 | Yes |
| 15 | SMURF1 | SMAD specific E3 ubiquitin protein ligase 1 [Source:HGNC Symbol;Acc:HGNC:16807] | 12344 | -0.688 | 0.0431 | Yes |
Table: GSEA details [plain text format]

  

Fig 2: GOMF\_ACTIVIN\_BINDING      
 Blue-Pink O' Gram in the Space of the Analyzed GeneSet

  

Fig 3: GOMF\_ACTIVIN\_BINDING: Random ES distribution      
 Gene set null distribution of ES for **GOMF\_ACTIVIN\_BINDING**
